# IgG Antibodies Generation and Side Effects Caused by Ad5-nCoV Vaccine (CanSino Biologics) and BNT162b2 Vaccine (Pfizer/BioNTech) among Mexican Population

**DOI:** 10.3390/vaccines9090999

**Published:** 2021-09-08

**Authors:** Oscar Guzmán-Martínez, Kathia Guardado, Elsa Ladrón de Guevara, Saturnino Navarro, Crescencio Hernández, Roberto Zenteno-Cuevas, Hilda Montero

**Affiliations:** 1 Instituto de Salud Pública, Universidad Veracruzana, Xalapa 91190, Mexico; oscarguzmanmtz@yahoo.com (O.G.-M.); kathia.aguardado@gmail.com (K.G.); elsaladron@uv.mx (E.L.d.G.); rzenteno@uv.mx (R.Z.-C.); 2 Centro de Investigaciones Biomédicas, Universidad Veracruzana, Xalapa 91190, Mexico; 3 Facultad de Medicina, Universidad Veracruzana, Xalapa 91010, Mexico; snavarro@uv.mx (S.N.); cosorio@uv.mx (C.H.)

**Keywords:** SARS-CoV-2, vaccines, BNT162b2, Ad5-nCoV, antibodies, adverse effects

## Abstract

SARS-CoV-2 has rapidly generated a pandemic. Vaccines are currently being rolled out to control the viral spread and prevent deaths. Emergency vaccines, using new platforms, have been approved. Their effectiveness, safety and immunogenicity in different populations are not fully known. This study aimed to discover the immunogenicity of the messenger ribonucleic acid (mRNA) BNT162b2 and adenovirus vector Ad5-nCoV vaccines through IgG antibody generation against subunit 1 of protein S (S1 IgG) and assess the side effects of the vaccines. A total of 115 vaccinated people were included, 61 of whom received the BNT162b2 vaccine, while 54 received Ad5-nCoV. Measurements of S1 IgG antibodies were carried out using the enzyme-linked immunosorbent assay (ELISA) technique. The BNT162b2 vaccine generated S1 IgG antibodies in 80.3% of the participants after the first dose. The number of seropositive participants increased to 98.36% with the administration of the second dose. The Ad5-nCoV vaccine generated S1 IgG antibodies in 88.89% of those vaccinated. Women generated more antibodies when administered either vaccine. There were no serious adverse effects from vaccination. In conclusion, not all participants had detectable S1 IgG antibodies. The Ad5-nCoV vaccine presented the most seronegative cases. The studied vaccines were shown to be safe.

## 1. Introduction

SARS-COV-2 has recently been circulating in humans and generating the COVID-19 disease, with diverse clinical manifestations, from asymptomatic infection to severe forms of the disease [1]. In addition to having caused many deaths worldwide [2], it was reported that the sequelae of the infection are varied and could be long-lasting; this is known as post-COVID-19 syndrome [3]. In the current situation, good hygiene and social distancing measures have had a positive impact on the control of viral spread; however, one of the most important public health measures that could help control the pandemic is vaccination [4].

The use of messenger ribonucleic acid (mRNA) that codes for the spike protein (protein S) of SARS-CoV-2 and the use of adenovirus vectors that carry the gene that codes for the same viral protein are new-generation platforms of vaccines that were approved for emergency use against SARS-CoV-2 [5]. Published studies of vaccines suggest that they are immunogenic, effective and well-tolerated [6,7,8,9,10,11]. However, their effectiveness is still being evaluated and could differ according to the population and the circulating variants, which could evade the protection conferred by vaccination [12,13,14]. It is important to note that the protection of vaccines authorized for emergency use against SARS-CoV-2 is from severe COVID-19 and not necessarily from infection [15].

In Mexico, some vaccines against COVID-19 were approved [16]. Regarding the new platforms, to date, an mRNA vaccine called BNT162b2 (Pfizer/BioNTech) and three that use viral vectors—AZD1222 (AstraZeneca), Sputnik V (Gamaleya Research Center) and Ad5-nCoV (CanSino Biologics)—have been approved. The vaccines require two doses, except Ad5-nCoV, which requires only one dose. The researchers behind the BNT162b2 vaccine reported the results of three phases of clinical trials [6,7,8,9]; however, those behind the Ad5-nCoV vaccine have, so far, only published the first two phases [10,11]. The Mexican authorities designed a vaccination plan for age groups and groups considered priorities, such as medical and educational personnel [17]. The selection of the vaccine to be applied is based on availability, except for the educational sector, in which the Ad5-nCoV vaccine is exclusively offered.

There is little knowledge of the immunogenicity, side effects and effectiveness of vaccines that use new platforms; generating knowledge is important for the follow-up of those vaccinated and for health authorities’ decision-making. In this study, to investigate immunogenicity, the generation of IgG memory antibodies that confer long-term seroprotection was evaluated. The IgGs antibodies for subunit 1 of protein S (S1 IgG) in SARS-CoV-2 were measured after the application of the BNT162b2 and Ad5-nCoV vaccines. In addition, the side effects of vaccination and some additional factors associated with this generation of IgG antibodies were evaluated.

## 2. Materials and Methods

### 2.1. Study Population

The inclusion criteria were persons over 18 years of age receiving the BNT162b2 or Ad5-nCoV vaccine. The exclusion criterion was COVİD-19 infection between vaccination doses. After signing the informed consent, peripheral blood samples were taken to obtain serum, which were stored at −80 °C until analysis. Two groups were formed—the first comprised those vaccinated with BNT162b2, from whom a sample was taken between three and four weeks after the first dose and a second sample two to three weeks after the second dose—based on previous published studies with this vaccine [6,7,9]. In the second group, participants vaccinated with Ad5-nCoV were sampled five to six weeks post-vaccination, with the intention that this was a similar timeframe to the full schedule of the BNT162b2 vaccination. This study was carried out in the city of Xalapa, Veracruz, Mexico from April to June 2021. The study was approved by the Research Ethics Committee of the Public Health Institute of the Universidad Veracruzana, with registration number CEI -ISP-R03/2020-E01/2021.

### 2.2. S1 IgG Antibodiesdetection

A commercial enzyme-linked immunosorbent assay (ELISA) kit was used for the detection of S1 IgG of SARS-CoV-2, according to the manufacturer’s specifications (EUROIMMUN Medizinische Labordiagnostika, Lübeck, Germany). The kit is authorized by the United States Food and Drug Administration (FDA) for emergency use [18]. The results were expressed semi-quantitatively by means of the index, dividing the value of the optical density (OD) of each serum (average of the duplicate) by the value of the OD calibrator. According to the manufacturer, values equal to or greater than 1.1 are considered positive. The plates were read at a measurement wavelength of 450 nm and a reference wavelength of 620 nm, in a Multiskan FC reader (Thermo Fisher Scientific, Vantaa, Finland).

### 2.3. Statistical Analysis

For data analysis, the SPSS version 25 program was used. For categorical variables, graphs, frequency tables and crossed tables were constructed. Descriptive statistics were calculated for quantitative variables, such as the mean, standard deviation, median, variance, box plots, scatter plots and Pearson’s correlation. For comparison of means, the Student’s t-test statistic was used for independent and dependent samples.

## 3. Results

### 3.1. Study Population

In this study, there was a sample of 115 participants; 53% (61) of them received the BNT162b2 vaccine and 47% (54) the Ad5-nCoV vaccine. The global average age was 55.9 ± 15.3 years. In the group vaccinated with BNT162b2, 75.4% (46/61) were women and 24.6% (15/61) men (Table 1). The mean age for the group vaccinated with BNT162b2 was 68.6 ± 6.18 years; the mean age of the men was 70.86 ± 6.73 years, while for women, the average age was 67.85 ± 5.88 years. In the group that received the Ad5-nCoV vaccine, 53.7% (29/54) were women and 46.3% (25/54) were men. The mean age was 41.5 ± 8.02 years; the mean age of the men was 42.36 ± 9.03 years and that of the women was 40.69 ± 7.12 years.

The most frequent comorbidities in the BNT162b2 vaccine group were arterial hypertension (39.3%), followed by being overweight or obese (24.6%). In the Ad5-nCoV vaccine group, the comorbidities presented were being overweight or obese (22.2%), followed by allergies in 20.4% of the participants (Table 1).

### 3.2. Detection of S1 IgG Antibodies

**Figure 1:**
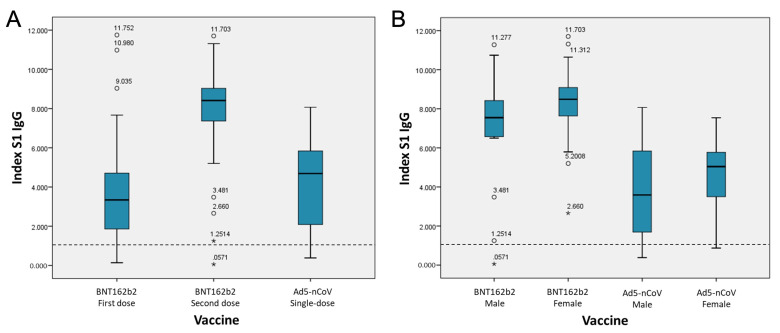

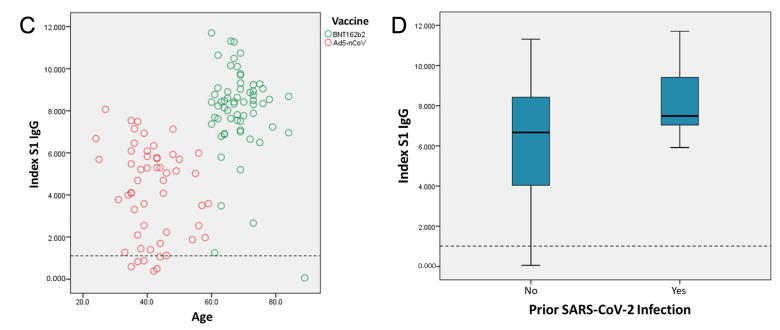
Values of S1 IgG indices by BNT162b2 and Ad5-nCoV vaccination, based on different conditions. (**A**) Indices generated by type of vaccine. (**B**) Indices generated by gender. (**C**) Correlation of index values and age. (**D**) Indices in persons with and without previous COVID-19, for both vaccines studied. ◦ Outliers: cases with the values between 1.5 and 3 box-lengths from the 75th percentile or 25th percentile. * Extreme values cases with the values more than 3 box-lengths from the 75th percentile or 25th percentile. Dashed horizontal line indicates the cut-off point; values equal or greater than 1.1 are considered positive.

The ELISA technique was used to determine the number of S1 IgG antibodies generated in each participant. The technique yields semi-quantitative values through an index; indices greater than 1.1 are considered positive. Regarding the detection of S1 IgG antibodies, of the participants who received the BNT162b2 vaccine, 19.7% did not detect S1 IgG antibodies after the application of the first dose. The percentage of participants in whom S1 IgG was not detected at the second dose decreased to 1.64%. On the other hand, with the Ad5-nCoV vaccine, S1 IgG antibodies were not detected in 11.11% of the participants. Regarding the number of antibodies generated, after the first dose with the BNT162b2 vaccine, S1 IgG indices were presented, with a median of 3.56 ± 2.53, rising to 8.01 ± 2.11 after the application of the second dose, a statistically significant increase (*p* = 0.000). The Ad5-nCoV vaccine had a mean S1 IgG index of 4.19 ± 2.18 (Figure 1A).

With the BNT162b2 vaccine, women were associated with higher production of S1 IgG antibodies when compared to men, with a mean of 8.37 ± 1.55 versus 6.90 ± 3.11 for men (*p* = 0.010) (Table 2, Figure 1B). In the same way, the application of the Ad5-nCoV vaccine generated higher rates in women (4.53 ± 1.84) compared to men (3.79 ± 2.49; *p* = 0.027). A correlation analysis was performed between S1 IgG indices and age. The general correlation between the value of the S1 IgG index and age was 0.527. The correlation analysis of each vaccine in its complete scheme was −0.199 for the BNT162b2 vaccine, while the correlation found with the application of the Ad5-nCoV vaccine was −0.186 (Figure 1C).

To find out if certain comorbidities or gender were statistically associated with the generation of antibodies, the Student’s t-test was applied. For the analysis, the values of the S1 IgG indices and the complete schedules of each vaccine were used. Of the comorbidities found in the study population, the participants with immunosuppression presented lower levels of antibodies when they were vaccinated with BNT162b2 (*p* = 0.018) (Table 2). No statistical significance was found with the other comorbidities.

People with a history of COVID-19 had the highest levels of S1 IgG antibodies with both vaccines. For the BNT162b2 vaccine, participants without prior COVID-19 had a mean of 7.89 ± 2.05, while with previous COVID-19, the mean was 11.49 ± 0.3. For the participants that received the Ad5-nCoV vaccine, the mean index in people without previous COVID-19 was 3.90 ± 2.07, while with previous COVID-19, it was 7 ± 0.65. Due to the small number of cases, no statistical test was applied. Figure 1D shows the means obtained from the S1 IgG indices of both vaccines, with and without COVID-19 before vaccination.

### 3.3. Side Effects of Vaccination

The secondary effects that occurred in the participants after the application of the vaccines were evaluated. After the first dose of BNT162b2, 68.9% of the participants experienced side effects, and the number of participants with side effects increased to 72.1% with the application of the second dose. With the first dose of BNT162b2, the most reported side effects were pain at the vaccination site (63.9%) and fatigue (24.6%). After the application of the second dose, pain at the vaccination site remained one of the most reported effects in 59% of participants, followed by fatigue in 32.8%. Of the participants vaccinated with Ad5-nCoV, 81.5% experienced side effects. The effects reported with this vaccine were fatigue in 50% of participants, pain at the vaccination site in 48.1%, muscle pain in 35.2% and a headache in 25.9% (Table 3). No serious adverse effects were reported with any of the vaccines.

## 4. Discussion

Throughout the period in which the SARS-CoV-2 virus became a permanent threat to the health and life of the population [19], scientific knowledge, publications, government commitments, major public programs and the concerns of most of the threatened population increased as never before. Despite this, there are still many unanswered or partially answered questions. In an incredible period, many vaccines were developed [20], which provided hope of finding a way to recover from this pandemic in the absence of an effective therapeutic resource. Some of these developed vaccines use new platforms, and their effectiveness will become known only as they are applied to the population. One of the ways to test the response generated by a vaccine is to study the generation of antibodies [21]; accordingly, serological studies must be carried out in various populations.

In this study, two new-platform vaccines were studied: BNT162b2 and Ad5-nCoV. The generation of S1 IgG antibodies as a result of these vaccines was evaluated. In the case of the BNT162b2 vaccine S1 IgG antibodies were detected after the application of the first dose in 80.3% of the participants. With the application of the second dose, the seroconversion percentage increased to 98.36%. This result is consistent with the fact that the pharmacist indicates a two-dose vaccination schedule [22] and highlights the importance of applying both doses. Regarding the Ad5-nCoV vaccine, 88.89% of the participants generated detectable S1 IgG antibodies. Both vaccines were reported to generate cellular and humoral responses [6,7,8,9,10,11], and both immune responses were reported to be important for the resolution of infection. Regarding the importance of humoral immunity, in animal models, studies show that the generation of IgG and neutralizing antibodies is important to defend against SARS-CoV-2 infection [23,24]. The media reported mortality from COVID-19 in people vaccinated by BNT162b2 and Ad5-nCoV in Mexico without having official data [25]. In other countries, mortality from COVID-19 was also reported in vaccinated people [26]. Although this study sets the precedent that not everyone who receives a SARS-CoV-2 vaccine has detectable S1 IgG antibodies, it has not yet been studied whether vaccination-seronegative people are those most affected when they become infected with SARS-CoV-2. The finding of this work is important as it suggests that the response expected from vaccination at the level of the generation of IgG antibodies does not occur in all cases, and this could vary between vaccines and in different populations. This topic is of high importance and should be studied in greater detail as a priority topic.

In the evaluation of vaccines, the number of antibodies generated, as well as their quality, are assessed through the measurement of neutralizing antibodies [27]. The methods for measuring antibodies have varied between the studies reported for different vaccines against SARS-CoV-2. In this work, the ELISA technique was used to detect IgG antibodies for the S1 protein; it was reported that the kit used in this study is effective in the detection of neutralizing antibodies [28,29]. In addition, we know the values of S1 IgG indices of people who have had COVID-19 (manuscript in preparation), which allows us to make a comparison with the values obtained by vaccination. The BNT162b2 vaccine, in its complete scheme, generated a higher number of S1 IgG antibodies (mean 8.01) compared to the Ad5-nCoV vaccine (mean 4.19), although the differences are not statistically significant (*p* = 0.076). The BNT162b2 vaccine generated very similar numbers of antibodies to those found in people with severe COVID-19 (median of 8.22; manuscript in preparation), although some participants achieved higher numbers of antibodies than those found in people with the natural infection. Participants vaccinated with Ad5-nCoV had lower antibody levels than those who had asymptomatic SARS-CoV-2 infection (median 6.4; manuscript in preparation), determined with the same test. The number of antibodies necessary to confer seroprotection is not known; however, a higher generation of antibodies is associated with a longer duration of seroprotection [30] and greater protection against circulating SARS-CoV-2 variants [31].

In the population of this study, women presented higher levels of S1 IgG antibodies after receiving either vaccine, contrary to the response observed in men, who generated higher numbers of antibodies due to SARS-CoV-2 infection [32,33]. Regarding age, our two groups (one per vaccine) showed very different age averages. This was the case because, in the city of the study, the health authorities indicated the application of the BNT162b2 vaccine in the population group aged 60 years and over, while the Ad5-nCoV vaccine was indicated for the entire educational sector, which includes a population with a wide age range. It should also be noted that, for the group who were over 60 years old, the BNT162b2 vaccine was applied before the Ad5-nCoV vaccine; it was for this reason that there were no people over 60 years old in the second group.

Although the correlation of age with the number of antibodies generated (i.e., the older the age, the lower the number of antibodies) was negative for both groups in this study (i.e., those vaccinated with BNT162b2 and Ad5-nCoV), it is important to carry out a study in a group of people with similar ages to be able to corroborate the observed pattern, since it was reported that age could influence the generation of antibodies [34]. If age is an important factor in the generation of antibodies, the seroconversion of Ad5-nCoV in older people should be studied since, in this study, the Ad5-nCoV vaccine presented a lower percentage of seroconversion in younger people.

Vaccines can generate secondary effects due to their application and, therefore, the safety of vaccines must be evaluated. Studies of the BNT162b2 and Ad5-nCoV vaccines show that they are safe [6,7,8,9,10,11,21]. In this work, some of the possible side effects of the application of the vaccines under study were evaluated. The side effects found in the population that received the first and second doses of the BNT162b2 vaccine corresponded to those reported in phase-three trials [7]. With respect to those who received the Ad5-nCoV vaccine, there was a higher percentage of cases with redness, fatigue, fever, nausea, headaches and muscle pains than was reported by the phase-two clinical trial [10]. No serious side effects were found in the groups of participants in this study.

In general, the BNT162b2 and Ad5-nCoV vaccines were shown to be well-tolerated and generated an S1 IgG antibody response in most participants. Vaccine non-responders should be studied in greater detail to find necessary answers to strengthen or redirect national vaccination policies.

## Figures and Tables

**Table 1:** General characteristics of the study population.

Characteristic	Vaccine				Total	
	BNT162b2		Ad5-nCoV		*n* = 115	%
	n	%	n	%		
**Sex**						
Male	15	24.6	25	46.3	40	34.8
Female	46	75.4	29	53.7	75	65.2
**Comorbidities**						
Diabetes	11	18.0	3	5.6	14	12.2
Arterial hypertension	24	39.3	2	3.7	26	22.6
Cancer	3	4.9	0	0	3	2.6
Allergies	13	21.3	11	20.4	24	20.9
Chronic kidney disease	1	1.6	0	0	1	0.9
Chronic liver disease	1	1.6	0	0	1	0.9
Immunosuppression	2	3.3	2	3.7	4	3.5
Obesity and overweight	15	24.6	12	22.2	27	23.5
Other chronic disease	11	18.0	3	5.6	14	12.2

**Table 2:** Characteristics associated with the generation of post-vaccination anti-S1 IgG antibodies, as measured by indices.

Characteristic	Vaccine							
	BNT162b2 (Second Dose)				Ad5-nCoV			
	Mean	SD	*n* = 61	*P*	Mean	SD	*n* = 54	*P*
Comorbidities								
**Diabetes**								
Yes	6.93	3.00	11	0.095	2.86	2.31	3	0.883
No	8.25	1.82	50		4.26	2.16	51	
**Arterial hypertension**								
Yes	7.99	2.02	24	0.391	1.5	0.54	2	0.072
No	8.02	2.20	37		4.49	2.15	52	
**Cancer**								
Yes	8.55	1.05	3	0.463	0	0	0	-
No	7.98	2.16	58		4.19	2.18	54	
**Allergies**								
Yes	7.99	1.02	13	0.102	3.21	2.24	11	0.915
No	8.01	2.34	48		4.44	2.12	43	
**Immunosuppression**								
Yes	5.06	5.38	2	0.018	3.1	2.89	2	0.819
No	8.11	1.96	59		4.23	2.17	52	
**Obesity and overweight**								
Yes	8.36	1.31	15	0.169	3.85	2.38	12	0.44
No	7.89	2.32	46		4.29	2.14	42	
**Sex**								
Female	8.37	1.55	46	0.010	4.53	1.84	29	0.027
Male	6.90	3.11	15		3.79	2.49	25	

A comparison of means was performed using the t-Student for independent and dependent samples. SD: Standard deviation.

**Table 3:** Side effects post-vaccination.

Side Effect	Vaccine					
	BNT162b2				Ad5-nCoV	
	First Dose		Second Dose		Single-Dose	
	*n*	%	*n*	%	*n*	%
Injection site pain	39	63.9	36	59.0	26	48.1
Redness	1	1.6	5	8.2	2	3.7
Swelling	0	0	2	3.3	2	3.7
Fatigue	15	24.6	20	32.8	27	50.0
Fever	0	0	2	3.3	9	16.7
Nausea	0	0	3	4.9	4	7.4
Chills	1	1.6	2	3.3	7	13.0
Headache	5	8.2	4	6.5	14	25.9
Myalgia	5	8.2	11	18.0	19	35.2

## Data Availability

The data underlying this article will be shared on reasonable request to the corresponding author.
